# The Role of the *MYL4* Gene in Porcine Muscle Development and Its Molecular Regulatory Mechanisms

**DOI:** 10.3390/ani14091370

**Published:** 2024-05-02

**Authors:** Yourong Ye, Guoxin Wu, Haoqi Wang, Mengqi Duan, Peng Shang, Yangzom Chamba

**Affiliations:** 1 College of Animal Science, Tibet Agriculture and Animal Husbandry College, Linzhi 860000, China; yeyourong@xza.edu.cn (Y.Y.); wgx19981212@163.com (G.W.); 18239259327@163.com (H.W.); zduanduan0117@163.com (M.D.); 2 The Provincial and Ministerial Co-Founded Collaborative Innovation Center for R&D in Tibet Characteristic Agricultural and Animal Husbandry Resources, Linzhi 860000, China; 3 Key Laboratory for the Genetic Improvement and Reproduction Technology of the Xizang Swine, Linzhi 860000, China

**Keywords:** Tibetan pigs, *MYL4*, PSMSC, muscle growth and development

## Abstract

**Simple Summary:**

Muscle growth is essential for the economic sustainability of the swine industry. Gaining a deeper understanding of pig muscle development is crucial for improving both the quantity and quality of pork production. This study employed RNA-seq technology to conduct a transcriptomic analysis of the longissimus dorsi muscle (LD) in three different pig breeds with varying body sizes and growth rates: Tibetan pigs (TP), Wujin pigs (WJ), and large white pigs (LW). Significant differences in gene expression were observed among the different breeds. We identified that myosin light chain 4 (*MYL4*) plays a role in influencing muscle growth. Experimental validation confirmed that *MYL4* promotes skeletal muscle cell proliferation and inhibits degradation. These findings reveal the molecular mechanisms that control muscle growth and provide valuable insights for improving pig breeding practices, ultimately enhancing the efficiency and quality of meat production.

**Abstract:**

Muscle growth stands as a pivotal economic trait within pig production, governed by a complex interplay of multiple genes, each playing a role in its quantitative manifestation. Understanding the intricate regulatory mechanisms of porcine muscle development is crucial for enhancing both pork yield and quality. This study used the GSE99749 dataset downloaded from the GEO database, conducting a detailed analysis of the RNA-seq results from the longissimus dorsi muscle (LD) of Tibetan pigs (TP), Wujin pigs (WJ) and large white pigs (LW) at 60 days of gestation, representing diverse body sizes and growth rates. Comparative analyses between TPvsWJ and TPvsLW, along with differential gene expression (DEG) analysis, functional enrichment analysis, and protein–protein interaction (PPI) network analysis, revealed 1048 and 1157 significantly differentially expressed genes (*p* < 0.001) in TPvsWJ and TPvsLW, respectively. With stricter screening criteria, 37 DEGs were found to overlap between the 2 groups. PPI analysis identified *MYL5*, *MYL4*, and *ACTC1* as the three core genes. This article focuses on exploring the *MYL4* gene. Molecular-level experimental validation, through overexpression and interference of the *MYL4* gene combined with EDU staining experiments, demonstrated that overexpression of *MYL4* significantly promoted the proliferation of porcine skeletal muscle satellite cells (PSMSC), while interference with *MYL4* inhibited their proliferation. Furthermore, by examining the effects of overexpressing and interfering with the *MYL4* gene on the muscle hypertrophy marker *Fst* gene and the muscle degradation marker *FOXO3* gene, the pivotal role of the *MYL4* gene in promoting muscle growth and preventing muscle degradation was further confirmed. These findings offer a new perspective on the molecular mechanisms behind porcine muscle growth and development, furnishing valuable data and insights for muscle biology research.

## 1. Introduction

Pigs, as important domestic meat animals, also play a key role as models in human muscle disease research [1]. A deeper understanding of the growth and development of pig muscles is crucial both for scientific research and agricultural production. Different breeds of pigs show significant differences in muscle development, which directly affects the characteristics of the meat [2,3]. Tibetan pigs have excellent germplasm characteristics such as strong resistance to adversity, strong environmental adaptability, and good meat quality, but compared with the Wujin pig and the current stage of commercial production of the York pig, there is a slow growth rate and small size phenomenon [4,5]. Skeletal muscle development begins during the embryonic period, when specific genes such as *Pax* and *MRFs* promote the formation and differentiation of myogenic fibers. In the fetal period, myofibers continue to mature and increase in number, relying on nutritional and hormonal support. Postnatally, muscle adapts through use and environmental stimuli, and satellite cells support repair and regeneration [6,7]. Previous studies have shown that there is expression of the *MYL4* gene in embryonic skeletal muscle [8]. Therefore, revealing the complex regulatory mechanisms in the growth and development of pig muscle can not only promote the development of more effective breeding strategies and meat processing technologies [9] but also provide valuable biological insights for muscle disease research [10].

The *MYL4* gene encodes the alkaline light chain of myosin, predominantly expressed in the muscles and atria of animals during the embryonic stage. The protein produced by *MYL4* is an essential component of the myofilament complex [11]. Its expression product possesses Ca^2+^ binding and motor activity, participating in the structural components of skeletal muscle, and is associated with muscle development and sarcomere contraction [12]. The most current research on the *MYL4* gene focuses on cardiomyopathy [13,14]. However, with the continuous advancement of omics sciences, more evidence suggests the association of the *MYL4* gene with muscle growth and development [15]. The growth and development of skeletal muscle significantly influences an animal’s meat production capacity and meat quality [16,17]. Skeletal muscle satellite cells (PSMSCs) are the fundamental units of skeletal muscle formation [18,19]. Therefore, the cultivation of PSMSCs in vitro is crucial for advancing our understanding of the processes involved in pig muscle growth and development.

This study uses publicly available RNA-Seq data from the GEO database, performing differential analysis with the edgeR package R 4.3.1 on the sequencing results of RNA-Seq. Subsequent functional enrichment analysis and analysis of the PPI network of these differentially expressed genes (DEGs) identified the *MYL4* gene as a key candidate gene for the growth and development of pigs. Analysis of the protein structure of the pig *MYL4* gene using bioinformatics software Overexpression and interference of the *MYL4* gene were conducted in PSMSCs, and EDU staining proliferation experiments were used to verify the regulatory effect of the *MYL4* gene in myocytes. Additionally, the effects of overexpressing and interfering with the *MYL4* gene in PSMSCs on the expression levels of the muscle hypertrophy marker gene *Fst* and the muscle degradation marker gene *FOXO3* were examined. This research aims to investigate the role of the *MYL4* gene in the growth and development of pig muscles, revealing its critical function in regulating the expression of genes related to muscle growth and providing new perspectives on related issues in the field of biology.

## 2. Materials and Methods

### 2.1. Data Source

Data for this study were obtained from publicly available RNA-Seq data in the Gene Expression Omnibus (GEO) database (https://www.ncbi.nlm.nih.gov/geo/ (accessed on 24 November 2023). Specifically, the GSE99749 dataset was downloaded from the GEO database. This dataset encompasses sequencing data for longissimus dorsi muscle tissue (LD) from three different breeds of pigs at 60 days of gestation: Tibetan pigs (TP), Wujin pigs (WJ), and large white pigs (LW).

### 2.2. Analysis of Differentially Expressed Genes

The GSE99749 dataset was analyzed for differential expression using the edgeR package R, (https://bioconductor.org/packages/edgeR/ (accessed on 24 November 2023). edgeR employs a model based on the negative binomial distribution to manage the variability in count data typical of sequencing data, making it particularly suitable for experimental designs with small sample sizes. This approach facilitates the identification of differentially expressed genes (DEG). On the basis of the results of the differential expression analysis, genes were ranked according to their *p*-values. Genes with *p* values less than 0.05 were considered to have statistically significant differential expressions.

### 2.3. Functional Enrichment Analysis

To explore the potential mechanisms of differentially expressed genes (DEGs) in pig muscle growth, gene ontology (GO) term annotations [20] and Kyoto Encyclopedia of Genes and Genomes (KEGG) pathway enrichment analyses [21] were performed using the OmicShare tools (https://www.omicshare.com/tools (accessed on 25 November 2023). A gene or pathway is considered statistically significant if both the False Discovery Rate (FDR) and *p*-value are less than 0.05 [22].

### 2.4. Screening of Key Candidate Genes

To further enhance the accuracy and reliability of differentially expressed genes (DEGs), more stringent filtering was applied to the DEGs between TP vs. WJ and TP vs. LW. The selection criteria were established as an absolute value of |log2FC| (fold change) < 8, along with a false discovery rate (FDR) of less than 0.001. For the conversion of gene ID, the DAVID online platform (https://david.ncifcrf.gov/summary.jsp (accessed on 25 November 2023) was used. Subsequently, online software STRING 12.0 (https://string-db.org/ (accessed on 25 November 2023) was used for protein–protein interaction (PPI) analysis, thereby identifying key candidate genes.

### 2.5. Animals

In this experiment, 3 Tibetan pigs (TP) and 3 large white pigs (LW) were selected from the Animal Science Teaching and research field of Tibet Agricultural and Animal Husbandry College. All pigs were healthy, castrated males aged 6 months, with no family connection within each group. Longissimus dorsi muscle tissues (LD) were collected from eight pigs of each breed, rapidly frozen in liquid nitrogen, and stored at −80 °C for RNA extraction.

### 2.6. RNA Extraction and Fluorescence Quantitative PCR

Total RNA was extracted from the LD tissues using Trizol reagent (Thermo Fisher, Waltham, MA, USA). The mRNA sequences for the porcine *MYL4*, *Fst*, *FOXO3* and *β-actin* genes were downloaded from the NCBI database. The primers for quantitative fluorescent PCR were designed using Primer Premier 5.0 software (Table 1) and synthesized by Shanghai Biotechnology Corporation (Shanghai, China). cDNA was prepared using a reverse transcription kit (Tiangen Biotech Co., Ltd., Beijing, China). The resulting cDNA served as the template, with three replicate wells set for each sample. A 20 μL PCR reaction system and protocol were used for the detection of fluorescent PCR in real time of the respective target genes and the internal reference gene. Gene expression levels were calculated using the 2^−ΔΔCt^ method.

### 2.7. Prediction of Protein Structure Properties of the MLY4 Gene

To predict various structural characteristics of the protein encoded by the *MYL4* gene, the amino acid sequence of the *MYL4* protein (Protein ID: XP_020922453.1) was downloaded from the NCBI database (http://www.ncbi.nlm.nih.gov/ (accessed on 27 November 2023): mppkkpepkk eaakaapapa papapapapp pepakeptfd pksikidfta dqieefkeaf slfdrtptge mkitygqcgd vlralgqnpt naevlrvlgk pkpeemnakm ldfetflpil qhisrnkeqg tyedfveglr vfdkesngtv mgaelrhvla tlgekmteae veqllagqed angcinyeaf vkhimsg. Subsequently, the secondary structure of the protein was predicted by logging into SOPMA (https://npsa-prabi.ibcp.fr/ (accessed on 27 November 2023), and its tertiary structure was predicted using the online software SWISS-MODEL (https://swissmodel.expasy.org/ (accessed on 27 November 2023).

### 2.8. PSMSC Cell Culture

PSMSC cells, acquired from Shanghai Cell Bank, were cultured in DMEM medium supplemented with 10% fetal bovine serum (FBS) and a solution of 2% penicillin-streptomycin (PS). After centrifugation (1000 r/min, 5 min), cells were resuspended in fresh medium and seeded in T25 flasks, then incubated at 37 °C with 5% CO_2_. When cell growth reached approximately 90% confluency, they were washed with 2 mL of phosphate buffer saline (PBS), treated with 0.25% EDTA-trypsin, and after centrifugation, resuspended in fresh medium. The cells were then transferred to new flasks in a 1:3 ratio for further culture.

### 2.9. Plasmid Construction, SiRNA Design, and Transfection

Based on the wild boar *MYL4* gene mRNA sequence published in the NCBI website GenBank (XM_021066794.1), seamless cloning primers for the sequence of the coding region of the *MYL4* gene were designed using the Tiangen seamless cloning primer online website (http://123.56.75.195/ (accessed 30 November 2023). The EcoRI restriction enzyme was selected for the cleavage site, with the upstream and downstream primer sequences being MYL4-F: 5′-tagtccagtgtggtggaattcATGCCTCCCAAGAAGCCTGA-3′ and MYL4-R: 5′-tgctggatatctgcagaattcTCACCCAGACATGATGTGCTTG-3′ (uppercase letters represent specific amplification primers, lowercase letters represent homologous arms for the vector pcDNA3.1(+) and the underscore indicates the EcoRI restriction enzyme cleavage site). The sequence of the *MYL4* gene coding region in the pig was amplified using MYL4-F/MYL4-R as primers. The PCR reaction system (total 20 μL) included 0.5 μL of each primer, 10 μL PCR Mix, 8 μL RNase-Free H_2_O, and 1 μL cDNA template. The PCR amplification product and the vector pcDNA3.1(+), linearized by the EcoRI restriction enzyme, were connected using a homologous recombination mix and transformed into DH5α competent cells. The transformed cells were cultured overnight on LB agar plates containing ampicillin, and positive clones were identified by colony PCR.

The design and synthesis of siRNA were carried out by Shanghai Biotechnology Corporation, resulting in three pairs of siRNA and one pair of negative control (NC), with primer sequences listed in Table 2. Synthesized RNA oligos were dissolved in DEPC water to prepare 20 uM samples.

PSMSC cells were seeded in a 24-well plate and cultured for 24 h until the cell density reached 70~80%. Following the instructions of the Lipofectamine^®^ 2000 reagent kit(Thermo Fisher, Waltham, MA, USA), each well was supplemented with 500 ng of plasmid and 1.5 μL of LipofectamineTM 2000 reagent for cotransfection of cells and plasmids. The groups included a control group (transfected with pcDNA3.1(+)), an overexpression group (transfected with MYL4-pcDNA3.1(+)), a knockdown group (transfected with sscMYL4-186) and a negative control group (transfected with NC), with three biological replicates for each group. After 48 h, the cells were collected.

### 2.10. EDU Staining Verified the Expression of the MYL4 Gene in PSMSC

PSMSC cells were seeded in a 96-well plate at a density of 5 × 10^3^ cells per well to ensure a transfection density of 60~70% after 24 h. Following the protocol provided in the Lipofectamine^®^ 2000 reagent kit, the empty vector pcDNA3.1(+), the overexpression group, the negative control group, and the knockdown group were transfected into PSMSC cells. A total of 48 h after transfection, staining was performed according to the instructions of the EdU-488 kit (Beyotime Biotechnology, Shanghai, China). The cells were then washed with PBS. Photographic analysis of the cells was conducted using a fluorescent inverted microscope. Three replicate wells were set for each group, and the experiment was repeated three times.

### 2.11. Statistical Analysis

The differences in gene expression levels were assessed for statistical significance using IBM SPSS Statistics 26.0 software. Each pig breed had 3 bioreplicates, and each bioreplicate set included 3 technical replicates, performing *t*-tests to analyze the statistical differences between means. All values are presented as mean ± SEM (standard error of the mean). The results are represented by *p* values. A *p* value less than 0.05 indicates significant differences, less than 0.01 indicates highly significant differences, and greater than 0.05 indicates that there is no significant difference.

## 3. Results

### 3.1. Analysis of Differentially Expressed Genes

**Figure 1:**
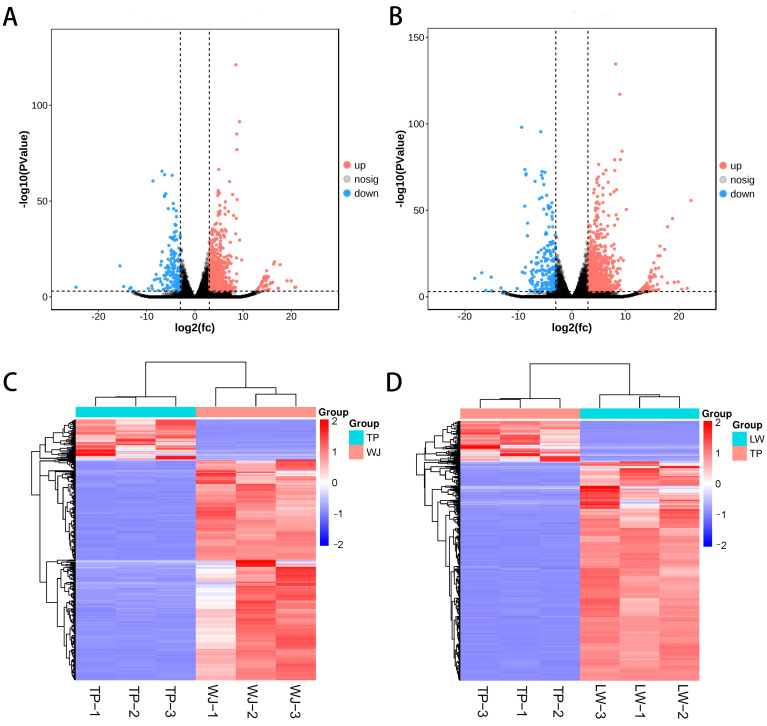
Differential gene expression (DEG) analysis. (**A**,**C**) Volcano plot and heat map of DEGs for TP vs. WJ. (**B**,**D**) Volcano plot and heatmap of DEGs for TP vs. LW.

The GSE99749 data in GEO database were analyzed using the edgeR software package R, revealing 1048 significantly differentially expressed genes (DEGs) between TP and WJ (*p* < 0.001), including 887 up-regulated DEGs and 161 down-regulated DEGs. Between TP and LW, there were 1371 DEGs (*p* < 0.001), with 1157 up-regulated DEGs and 214 down-regulated DEGs. In volcano plots, the x-axis represents the difference in fold change, denoted as |log2FC|. The larger the absolute value, the greater the fold change. The y-axis indicates the significance of the difference, represented as −log10(*p* value). The higher the value, the more significant the difference (Figure 1A,B). The expression distribution of DEGs is based on the clustering of gene expressions between samples (Figure 1C,D).

### 3.2. Functional Enrichment Analysis

**Figure 2:**
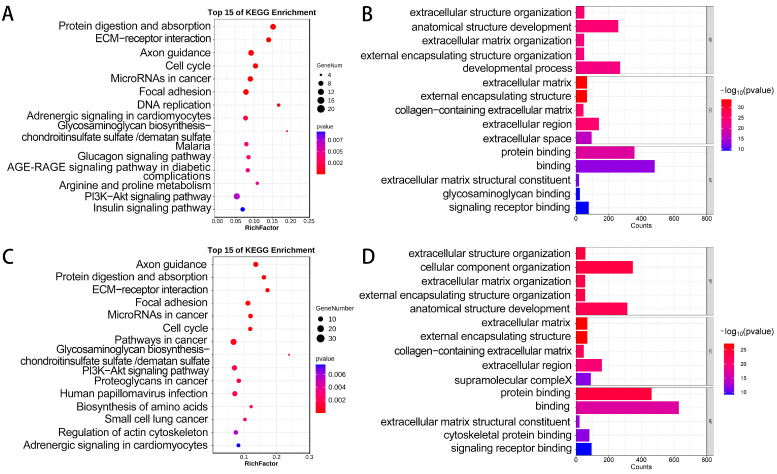
Functional enrichment results of DEGs. (**A**,**B**) GO and KEGG analyses for DEGs between TP and WJ. (**C**,**D**) GO and KEGG analyses for DEGs between TP and LW.

To further explore the potential biological functions of DEGs in porcine muscle growth, we conducted GO and KEGG enrichment analyses. The enrichment analysis of the KEGG pathway revealed that the DEGs between TP vs. WJ and TP vs. LW were mainly enriched in 15 pathways, including the PI3K-Akt signaling pathway, focal adhesion, and ECM-receptor interaction (Figure 2A,C). GO enrichment analysis indicated that, in terms of biological processes, DEGs were mainly enriched in the extracellular matrix organization, extracellular structure organization, and anatomical structure development. In the category of cellular components, DEGs were predominantly found in the extracellular matrix and the collagen-containing extracellular matrix. With respect to molecular functions, these DEGs focused mainly on protein binding and related aspects (Figure 2B,D).

### 3.3. Analysis of Key Candidate Genes

**Figure 3:**
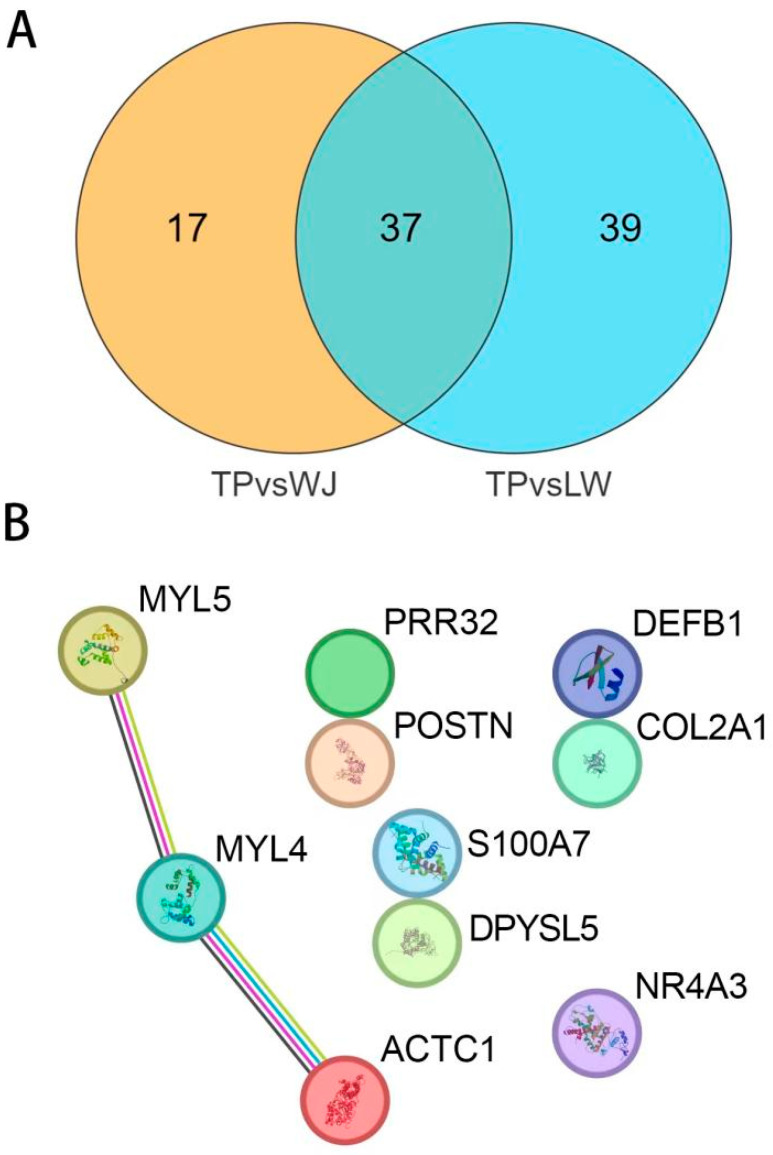
Venn diagram and protein–protein interaction (PPI) analysis of DEGs. (**A**) TP vs. WJ and TP vs. LW share 37 common DEGs. (**B**) The PPI results indicate that the *MYL4* gene interacts with both the *MYL5* gene and the *ACTC1* gene.

Based on filtering criteria: |log2FC| < 8 and FDR < 0.001, the dataset for TP vs. WJ contained 54 DEGs that met these requirements, while the dataset TP vs. LW had 76 DEGs. Through Venn diagram analysis (Figure 3A), we identified 37 differentially expressed genes (DEGs) that overlap between the two groups. When converting these DEGs’ gene identifiers (IDs) to gene names, only 10 gene IDs could be successfully matched to their respective gene names (Table 3). For these 10 identified genes, we further constructed a protein–protein interaction (PPI) network (Figure 3B). In this network, we identified three core genes, and the figure shows that the *MYL4* gene has a direct interaction with the *MYL5* gene and *ACTC1*, suggesting that the *MYL4* gene may have a more important role than the other two genes, and it is inferred that the *MYL4* gene may play a key role in the muscle growth process or the muscle development signaling pathway.

### 3.4. RT-qPCR Detection

**Figure 4:**
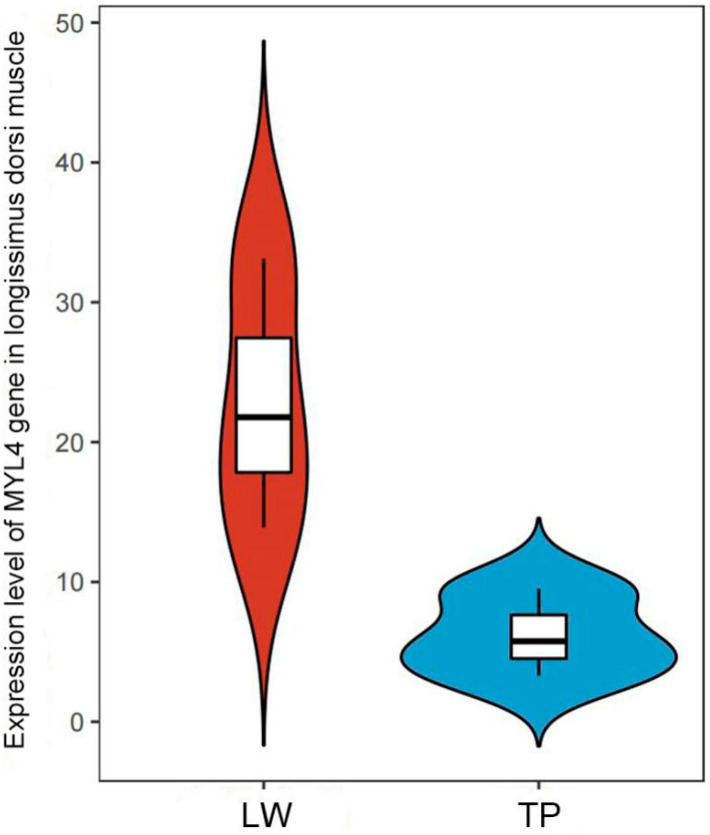
Fluorescent quantitative PCR results for the *MYL4* gene. LW refers to large white pigs, and TP refers to Tibetan pigs.

We compared the expression levels of the *MYL4* gene in the longissimus dorsi muscle (LD) between Yorkshire and Tibetan pigs. As shown in Figure 4, the results indicate that the expression level of the *MYL4* gene in the longissimus dorsi muscle of Yorkshire pigs is significantly higher than in Tibetan pigs (*p* < 0.05).

### 3.5. Prediction of Secondary and Tertiary Structures of the MYL4 Protein

**Figure 5:**
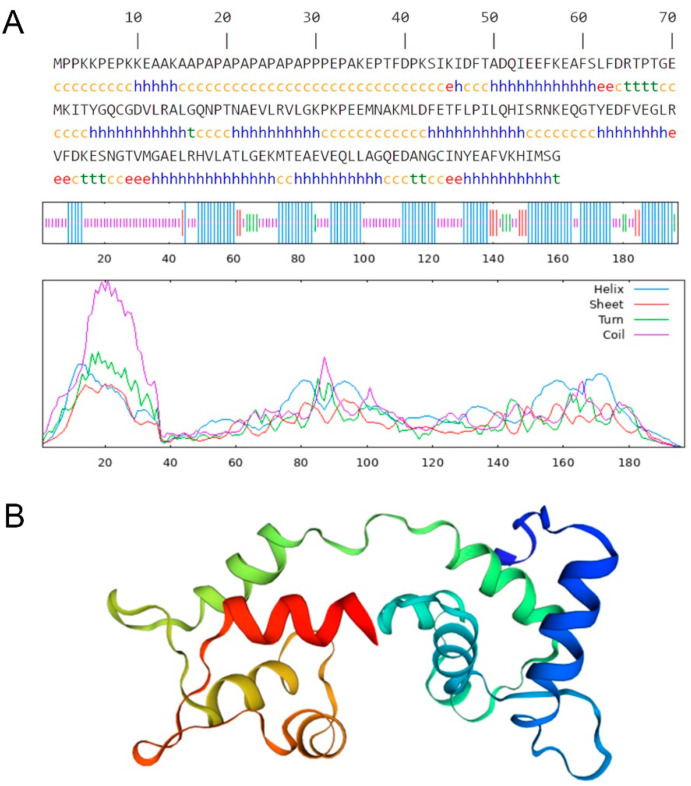
Prediction results of the protein structure of the *MYL4* gene. (**A**) Prediction of the secondary structure, with ‘h’ and blue lines representing α-helices; ‘e’ and red lines representing extended strands; ‘t’ and green lines representing β-turns; ‘c’ and purple lines representing random coil. (**B**) Predictive modeling of the tertiary structure.

The secondary structure of the porcine *MYL4* protein was predicted using the SOPMA software, with results shown in Figure 5A and Table 4. The analysis indicates that in the secondary structure of the porcine MYL4 protein, α-helices account for 46.70%; random coil for 42.13%; and both β-turns and extended strands constitute 5.58% each. The porcine MYL4 protein predominantly comprises α-helices and random coils, which may suggest that it has good elasticity and flexibility.

Through the online software SWISS-MODEL, it was predicted that the three-dimensional structure of the porcine MYL4 protein is predominantly composed of α-helices, aligning with the analysis of its secondary structure. This dominance of the α-helix structure suggests that the protein may rely on the elasticity and stability characteristics of α-helices to perform its biological functions (Figure 5B).

### 3.6. The Effect of Overexpression and Knockdown of the MYL4 Gene on PSMSC Proliferation

**Figure 6:**
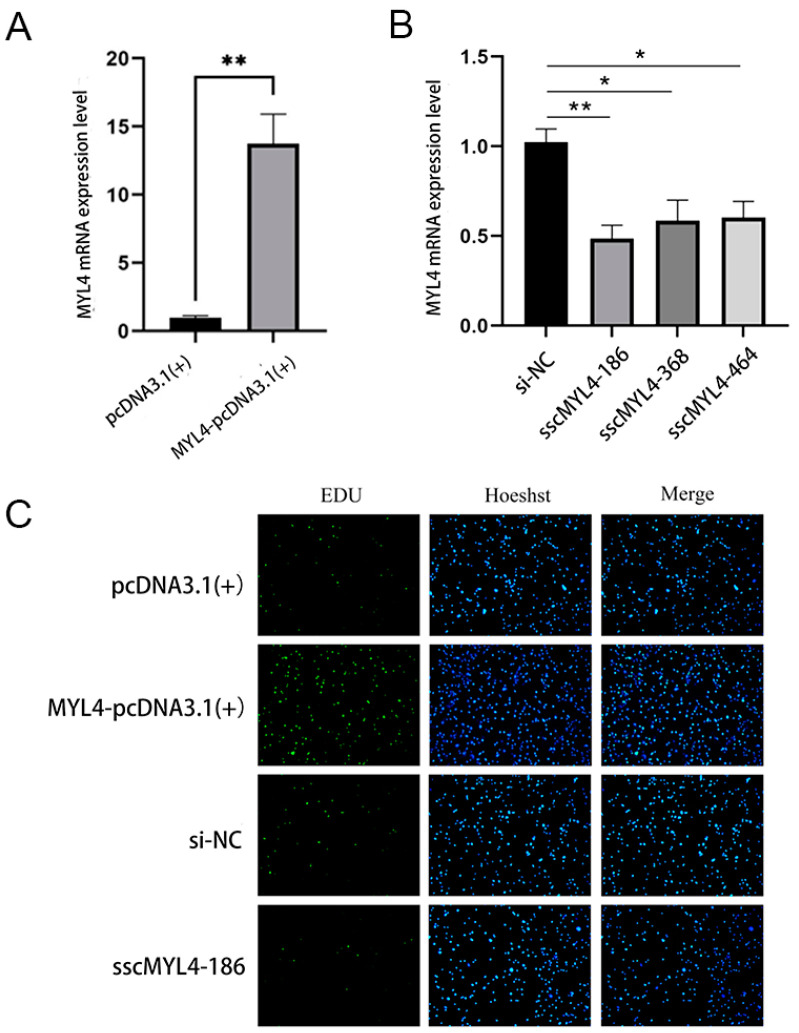
The impact of overexpressing and knockdown *MYL4* on PSMSC proliferation. (**A**) Results of RT-qPCR of overexpression of the *MYL4* gene. (**B**) RT-qPCR results of knockdown of the *MYL4* gene. (**C**) Proliferation results of EDU staining of transfected cells, where EDU marks the new cells, Hoeshst marks all cell nuclei, and Merge is the combination. * indicates significant difference *p* < 0.05, ** indicates highly significant difference *p* < 0.01.

The control group and the overexpression group were transfected into PSMSC cells. The results of RT-qPCR showed that the expression level of MYL4-pcDNA3.1(+) was significantly higher than that of pcDNA3.1(+) (*p* < 0.01) (Figure 6A), indicating that the overexpression vector was successfully overexpressed in PSMSC. The NC negative control group and three pairs of siRNAs were transfected into PSMSC cells, and the RT-qPCR results showed significant differences between the three pairs of siRNAs and the negative control group (Figure 6B). Based on the knockdown efficiency, sscMYL4-186 was selected for further experimental validation.

Control group A, overexpression group B, knockdown group C, and negative control group D were transfected into PSMSC cells and incubated for 48 h before being stained with EDU. The results of the EDU stain proliferation assay showed that after EDU labeling, the number of new cells in the overexpression group was significantly higher than in the control group, while the number of new cells in the knockdown group was significantly lower than in the negative control group (Figure 6C). These results indicate that overexpression of the *MYL4* gene can promote the proliferation ability of PSMSCs, while the suppression of the *MYL4* gene can inhibit the proliferation ability of PSMSCs.

### 3.7. The Impact of Overexpression and Knockdown of the MYL4 Gene on the Fst and FOXO3 Genes

**Figure 7:**
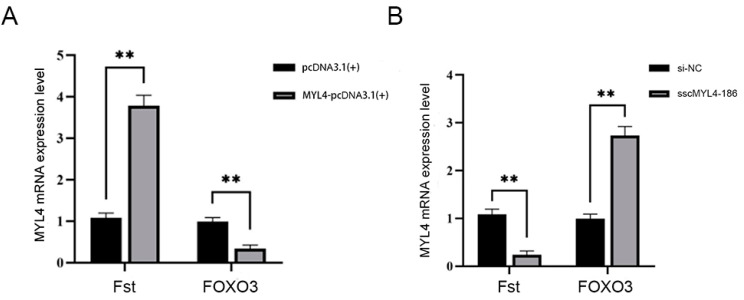
The impact of overexpression and knockdown of the *MYL4* gene on the *Fst* and *FOXO3* genes. (**A**) Overexpression of the *MYL4* gene promotes the expression of the *Fst* gene and inhibits the expression of the *FOXO3* gene. (**B**) Knockdown of the *MYL4* gene inhibits the expression of the *Fst* gene and promotes the expression of the *FOXO3* gene. ** indicates a highly significant difference *p* < 0.01.

The impact of overexpressing and knockdown of the *MYL4* gene on the mRNA expression levels of the *Fst* gene and the *FOXO3* gene, markers of muscle hypertrophy and muscle degradation, was studied. The results showed that after overexpression of the *MYL4* gene, the expression level of the *Fst* gene was significantly higher than that of the control group (*p* < 0.01), while the expression level of the *FOXO3* gene was significantly lower than that of the control group (*p* < 0.01). After removing the *MYL4* gene, the expression level of the *Fst* gene was significantly lower than that of the negative control group (*p* < 0.01), and the expression level of the *FOXO3* gene was significantly higher than that of the negative control group (*p* < 0.01) (Figure 7).

## 4. Discussion

The development of porcine skeletal muscle plays a crucial role in determining the growth rate of muscle and the quality of meat [23]. The *MYL4* gene encodes the myosin light chain, a basic component of the myosin complex in muscle, which is expressed primarily in the skeletal muscle during the embryonic stage of animals and in the atria of adult animals. Its expression product has Ca^2+^ binding and motor activity functions, and it is part of the structural components of muscle, related to muscle development and striated muscle contraction [24,25]. In this study, we downloaded the GSE99749 dataset from the GEO database to identify differentially expressed genes (DEGs) between LD tissues from TP, WJ, and LW pig breeds. Subsequent functional enrichment analyses of GO and KEGG were performed to explore the biological functions of DEG in muscle growth. The results of the enrichment of the KEGG pathway showed that DEGs were enriched primarily in pathways such as the PI3K-Akt signaling pathway, Focal adhesion, and ECM-receptor interaction. Research by Glass DJ [26] indicates that the PI3K/Akt signaling pathway promotes skeletal muscle hypertrophy by activating IGF-1 signaling. IGF-1 can inhibit the expression of muscle atrophy-related proteins MuRF1 and MAFbx (Atrogin-1), protecting muscle from degradation. Furthermore, the IGF-1/PI3K/Akt signaling pathway can also inhibit myostatin, thereby promoting muscle growth [27]. Myostatin commonly limits muscle growth by suppressing the Akt signaling pathway and inhibiting the differentiation of myoblasts. In terms of biological processes, DEGs were mainly enriched in the organization of extracellular structures, the development of anatomical structures and the organization of cellular components. Within the cellular components, DEGs were primarily focused on the extracellular matrix, encapsulating structures, and collagen-containing extracellular matrix. Regarding molecular functions, these DEGs were mainly involved in protein binding, among others. The extracellular matrix can influence intracellular signaling pathways in muscle cells through interactions with receptors on the surface of muscle cells, thus regulating muscle growth and repair [28]. By utilizing the STRING database to construct a protein–protein interaction (PPI) network, the *MYL4* gene was identified as a key candidate gene that could influence pig muscle growth. This finding aligns with the results of research conducted by Dong S [12] and colleagues. Using bioinformatics software to predict and analyze the structural characteristics of the *MYL4* gene protein, it was found that the porcine MYL4 protein is primarily composed of α-helices and random coils. This indicates that it possesses good elasticity and flexibility [29], making it well suited for functioning in complex biological environments, such as during muscle contraction and metabolic processes [30]. The advantage of its structure suggests that the protein may rely on the elasticity and stability characteristics of α-helices to perform its biological functions [31]. To further determine the differences in the *MYL4* gene between the TP and YY breeds, quantitative fluorescent PCR was used to examine the LD of TP and YY, showing that the expression level of the *MYL4* gene in the LD of YY was significantly higher than in TP. This suggests that the *MYL4* gene could be a key gene that promotes muscle growth and development.

PSMSCs not only serve as a source of nuclei within muscle fibers but also promote the hypertrophy of muscle fibers [32]. Activated SMSCs can induce muscle cell proliferation and differentiation and facilitate the fusion of muscle cells with muscle fibers, playing a vital role in muscle fiber regeneration and recovery processes [33]. Thus, PSMSCs can serve as an excellent in vitro model to explore the mechanisms of pig muscle growth and development. In this experiment, a homologous recombination method was used first to construct the overexpression vector MYL4-pcDNA3.1(+) and the designed and synthesized siRNA was transfected into PSMSCs. RT-qPCR was utilized to detect the efficiency of overexpression and interference. The results showed that the expression level of the *MYL4* gene was approximately four times higher than that of the control group, indicating a successful overexpression of the constructed vector in PSMSCs. Among the siRNAs, all three showed significant differences compared to the negative control group, and sscMYL4-186 was ultimately selected for further experimental validation based on interference efficiency. To verify the impact of the *MYL4* gene on the proliferative capacity of PSMSCs, four treatment groups were subjected to EDU staining. The results of the EDU staining proliferation assay revealed that, after EDU labeling, the number of new cells in the overexpression group was significantly higher than in the control group, while the number of new cells in the knockdown group was significantly lower than that of the negative control group. This indicates that overexpression of the *MYL4* gene can promote PSMSC proliferation, while interference with the *MYL4* gene can inhibit its proliferative ability. To further explore the effects of the *MYL4* gene on muscle cell hypertrophy and degradation, the muscle hypertrophy gene *Fst* [34] and muscle degradation marker gene *FOXO3* [35] were selected as reference genes. Fluorescent quantitative PCR results showed that after overexpressing the *MYL4* gene, the expression level of the *Fst* gene was significantly higher than in the control group, while the expression of the *FOXO3* gene was significantly lower than in the control group. After interference with the *MYL4* gene, the expression level of the *Fst* gene was significantly lower than in the negative control group, and the expression of the *FOXO3* gene was significantly higher than in the negative control group. These results suggest that the *MYL4* gene can promote muscle hypertrophy and inhibit muscle degradation. Through overexpression and siRNA techniques, the regulatory role of specific genes can be studied at the cellular level [36]. Our study helps to elucidate the roles of these genes in porcine muscle growth and development, and provides important clues for the study of the mechanism of porcine muscle growth and development. In the follow-up study, we can more accurately assess the muscle development growth status and health condition of pigs through regular detection of *MYL4* expression in pigs, thus adjusting the breeding management measures in a more targeted manner, and applying the discovery of *MYL4* to pig production.

## 5. Conclusions

In summary, using RNA-seq technology, we performed a comparative analysis of the longissimus dorsi muscle of pigs of three different body types and growth rates. This analysis identified the *MYL4* gene as a key candidate gene that affects the growth of pig muscles. Furthermore, our EdU staining experiments revealed that overexpression of the *MYL4* gene can enhance the proliferative capacity of PSMSCs, while interference with the *MYL4* gene inhibits proliferation of these cells. Furthermore, our experiments indicate that the *MYL4* gene plays a role in promoting muscle hypertrophy and preventing muscle degradation. These results provide important information on understanding the mechanisms of pig muscle growth and development. In addition, this finding may provide a basis for improving the overall health and welfare of animals. By enhancing muscle growth and slowing degradation, the meat quality and yield of animals could be improved, leading to potential economic benefits for agricultural production.

## Figures and Tables

**Table 1:** Specific primer sequences for the *MYL4* gene, the *Fst* gene, and the *FOXO3* gene.

Gene Name	Forward Primer (F)	Reverse Primer (R)
*MYL4*	AGGAACCCACCTTTGACCC	GCAGCACCTCGGCATTAG
*Fst*	TACCGCAACGAATGTGCTCT	TCTGGGCAAATGCGGTTACA
*FOXO3*	CAGCAGCACAGTGTTTGGAC	AGTGTCTGGTTGCCGTAGTG
*β-actin*	TCTGGCACCACACCTTCTA	AAGGTCTCGAACATGATCTG

**Table 2:** siRNA primer information.

Gene Name	Sense (5′–3′)	Antisense (5′–3′)
sscMYL4-186	GGAGAGAUGAAGAUCACCUTT	AGGUGAUCUUCAUCUCUCCTT
sscMYL4-368	GGGCACCUAUGAGGACUUUTT	AAAGUCCUCAUAGGUGCCCTT
sscMYL4-464	CCUGGGAGAGAAGAUGACUTT	AGUCAUCUUCUCUCCCAGGTT
NC	UUCUCCGAACGUGUCACGUTT	ACGUGACACGUUCGGAGAATT

**Table 3:** Information table for overlapping DEGs.

Gene ID	Gene Name	Universal Gene Name
ENSSSCG00000005385	*NR4A3*	nuclear receptor subfamily 4 group A member 3
ENSSSCG00000004803	*ACTC1*	actin alpha cardiac muscle 1
ENSSSCG00000006592	*S100A7*	S100 calcium binding protein A7
ENSSSCG00000009361	*POSTN*	periostin
ENSSSCG00000017307	*MYL4*	myosin light chain 4
ENSSSCG00000025523	*COL2A1*	collagen type II alpha 1 chain
ENSSSCG00000029990	*DEFB1*	defensin beta 1
ENSSSCG00000012647	*PRR32*	proline rich 32
ENSSSCG00000027502	*MYL5*	myosin light chain 5
ENSSSCG00000008560	*DPYSL5*	dihydropyrimidinase like 5

**Table 4:** Prediction results for the secondary structure of the MYL4 protein.

Structure Type	Proportion/%
α-helices (Hh)	46.70
Random coil (Cc)	42.13
β-turns (Tt)	5.58
Extended strands (Ee)	5.58

## Data Availability

Data for this study were obtained from publicly available RNA-Seq data within the Gene Expression Omnibus (GEO) database, accessible at (https://www.ncbi.nlm.nih.gov/geo/ (accessed on 24 November 2023)).
